# Transcriptome profiling of genes related to light-induced anthocyanin biosynthesis in eggplant (*Solanum melongena* L.) before purple color becomes evident

**DOI:** 10.1186/s12864-018-4587-z

**Published:** 2018-03-20

**Authors:** Jing Li, Yong-Jun He, Lu Zhou, Yang Liu, Mingmin Jiang, Li Ren, Huoying Chen

**Affiliations:** 1 School of Agriculture and Biology, Shanghai Jiao Tong University, 800 Dongchuan Road, Minhang District, Shanghai, 200240 China; 2 Institute for Agri-Food Standards and Testing Technology, Shanghai Academy of Agricultural Sciences, 1000 Jinqi Road, Fengxian District, Shanghai, 201403 China

**Keywords:** Eggplant (*Solanum melongena* L.), Transcriptome, Light, Anthocyanin biosynthesis, Before color becomes evident

## Abstract

**Background:**

The anthocyanins are highly enriched in eggplants (*Solanum melongena* L.) with purple peel. However, our previous study showed that anthocyanins biosynthesis in eggplant cultivar ‘*Lanshan Hexian*’ was completely regulated by light and color becomes evident at most 2 days after exposure to light. In the present investigation, transcriptome study was made to explore the underlying molecular mechanisms of light-induced anthocyanin biosynthesis in eggplant (*Solanum melongena* L.) before color becomes evident.

**Results:**

RNA-Seq was performed for four time points (0, 0.5, 4 and 8 h after bags removal) where concerted changes happened. A total of 32,630 genes or transcripts were obtained by transcriptome sequencing, from which 1956 differentially expressed genes (DEGs) were found. Gene Ontology analysis showed that the 1956 DEGs covered a wide range of cellular components, molecular functions and biological processes. All the DEGs were further divided into 26 clusters based on their distinct expression patterns. Kyoto Encyclopedia of Genes and Genomes (KEGG) pathway enrichment analysis found out 24 structural anthocyanin biosynthesis genes which distributing in seven clusters. In addition, 102 transcription factors, which exhibited highly dynamic changes in response to light, were found in the seven clusters. Three photoreceptors, UV Resistance Locus 8 (*UVR8*), Cryptochrome 3 (*CRY3*) and *UVR3*, were identified as DEGs. The light signal transduction elements, COP1 and two SPAs, might be responsible for anthocyanin biosynthesis regulation.

**Conclusion:**

Based on the transcriptome data, the anthocyanin biosynthesis structural genes, transcription factors, photoreceptors and light signal transduction elements were quickly screened which may act as the key regulatory factors in anthocyanin biosynthesis pathway. By comparing the transcriptome data with our previous studies, 869 genes were confirmed to participate in the light-induced anthocyanin biosynthesis. These results expand our knowledge of light-induced anthocyanin biosynthesis in plants, which allowing for fruit coloration to be improved under low-light conditions in future.

**Electronic supplementary material:**

The online version of this article (10.1186/s12864-018-4587-z) contains supplementary material, which is available to authorized users.

## Background

Anthocyanins are natural pigments and widely found in plants. They not only confer plants vivid colors to attract pollinators and seed dispersers, but also enhance plants resistance to stress [1]. Anthocyanins are synthesized from phenylalanine, and then under the continuous catalytic by phenylalanine ammonia lyase (PAL), 4-coumarate-CoA ligase (4CL), chalcone synthase (CHS), chalcone isomerase (CHI), flavanone 3-hydroxyl enzyme (F3H), dihydroflavonol reductase (DFR) and anthocyanin synthase (ANS) or leucoanthocyanidin dioxygenase (LDOX). This pathway creates unstable anthocyanins [2] that are stabilized by glycosylation (GT), methylation (MT) or acylation (AT) before transportation to the cell vacuole for storage [3]. Anthocyanins are mainly of six types: pelargonidin, cyanidin, delphinidin, peonidin, petunidin and malvidin [4]. The structural anthocyanin biosynthesis genes of many plant species have been identified and are mostly polygenes, such as *PAL*, *CHI*, *CHS*, and *DFR* [5–7].

These structural genes are simultaneously regulated by an activated transcriptional MBW complex, consisting of the R2R3-MYB and bHLH transcription factors, and the WD40 repeat protein [8, 9]. The MYB family is one of the largest gene families in plant. It is broadly divided into four sectors according to the number of conserved domains [10]. The R2R3-MYB transcription factors play a central role in anthocyanin biosynthesis through their influence on the transcriptional level of the structural anthocyanin biosynthesis genes [11, 12]. Most are positive regulators [e.g., *AtMYB75*, *PpMYB9* and *NnMYB5* [13–15]. However, others are inhibitory in the context of anthocyanin synthesis [e.g., *EsMYBF1*, *PtMYB182* and *VvMYB4* [16–18]. BHLHs belong to a multi-gene family that can be divided into 26 subgroups, and flavonoid related bHLHs were grouped into subgroup IIIf [19]. In *Arabidopsis*, some bHLHs, including TT8, GL3 and EGL3, have been identified as participants in the biosynthesis of different flavonoids [20–22]. In addition, the bHLHs which were associated with anthocyanin biosynthesis have been identified from a variety of plant species, such as CmbHLH2 [23], LcbHLH3 [24], MtbHLH (MtTT8) [25], LebHLH (AH) [26] and VvMYC1 [27]. Some other transcription factors, including Elongated Hypocotyl 5 (HY5), light-regulated zinc finger protein (LZF), CONSTANS-like (COL) and squamosa promoter binding protein-like 9 (SPL9), have also been shown to be associated with anthocyanin biosynthesis [28–31].

Light is a key environmental factor that affects the synthesis of anthocyanins [5, 32–36]. This process is facilitated by special photoreceptors. They transmit a signal to the downstream transcription factors that regulate plant growth and metabolic processes, including anthocyanin biosynthesis. The photoreceptors in higher plants have been identified to date including phytochromes (PHYs), phototropins (PHOTs), cryptochromes (CRYs) and UV-resistance locus 8 (UVR8). The light receptors are sensitive to specific wavelengths: PHYs detect red/far red, CRYs detect blue [37], PHOTs detect UV-A, and UVR8 detects the ultraviolet wavelengths [38]. The significance of the photoreceptors in anthocyanin biosynthesis has been confirmed by experiments with *Arabidopsis*. For example, *phyA* mutants could not induce the expression of *CHS* (an enzyme in the anthocyanin biosynthesis pathway) and the synthesis of anthocyanins under far red light [39]. The expression level of *CHS* and the content of flavonoids [40] were significantly reduced in the *uvr8* mutants. In addition, *CRY1* induced anthocyanins synthesis under blue light through the activation of anthocyanin biosynthesis enzymes [41].

Downstream of these photoreceptors, Constitutive Photomorphogenic 1 (COP1) acts as a central repressor in the anthocyanin biosynthesis pathway, including PAP1, PAP2, HY5, Hypocotyl Homolog (HYH), Long Hypocotyl in Far-Red 1 (HFR1) and Long After Far-Red Light 1 (LAF1) [42–47]. COP1 is considered to be a ‘molecular switch’ in metabolic processes which are stimulated by light [48]. Under dark conditions, COP1 was localized in the nucleus. COP1 promotes the degradation of the photomorphogenesis-promoting transcription factors [49]. Under light, the concentration of COP1 in the nucleus decreased rapidly [50] and the light-activated photoreceptors inhibited COP1 activities, thereby causing accumulation of the photomorphogenesis-promoting transcription factors. Maier et al. [46] reported that large amounts of anthocyanins were synthesized in *cop1* mutants under dark conditions. In addition, COP1 formed a complex with phytochrome A-105 (SPA) in the nucleus to inhibit photomorphogenesis [46, 51, 52]. In *Arabidopsis*, *spa* mutants exhibited similar features with COP1 mutants under dark conditions [53, 54].

Eggplant (*Solanum melongena* L.) is a commercially significant crop cultivated and consumed in many countries [55]. The varieties of eggplant with dark purple peel, which is caused by a high anthocyanin concentration, are more attractive to consumers than the paler types. The anthocyanin concentration in the purple eggplant cultivars is high in comparison to that of other deeply colored fruits and vegetables, e.g., 2.34× that of grapes*,* and 7.08× that of red onions [56].

The observation that anthocyanin biosynthesis is stimulated by light is paralleled in other species, e.g. lithci, Chinese bayberry, grape berry, apple, and red pear [6, 36, 47, 57]. Previous researches focused on exploring the regulatory mechanisms associated with the accumulation of anthocyanins at the molecular level [6, 7]. Recently, the effect of light on the whole process of anthocyanins biosynthesis was preliminary studied in our previous study [35]. However, the underlying molecular mechanisms of light-induced anthocyanins biosynthesis before color becomes evident are not well known.

Our previous study found out that anthocyanin biosynthesis in the peel of eggplant (cv *Lanshan Hexian*) is regulated by light and color becomes evident at most 2 days after exposure to light. [5, 35]. In this study, the expression patterns of a variety of anthocyanin biosynthesis structural genes and regulator genes under light over an 8 h period were analyzed by qRT-PCR. In the present study, RNA-seq was used to capture the majority of transcriptional changes in anthocyanin biosynthesis related genes. This analysis was attempted to elucidate the molecular mechanisms of light-induced anthocyanin biosynthesis before color becomes evident. This study will facilitate genetic engineering protocols which would improve the anthocyanin content of eggplant or other plants under low-light condition.

## Methods

### Plant materials and treatments

The eggplants were grown in the horticultural farm of Shanghai Jiao Tong University, Shanghai China. After full bloom, the sepals were covered with paper bags which were completely light-impermeable. The bags were removed from the fully-grown fruits after 24 days at 8:00 am. The peels of the eggplants were collected immediately after bags removal, and then after a further 0.5, 4 and 8 h. Three eggplant fruits were mixed as a sample and three samples were set as biological replicates. The pooled samples were immediately frozen in liquid nitrogen, and stored at − 80 °C prior to further processing.

### RNA extraction, library construction and transcriptome sequencing

Total RNA was extracted from pooled samples using RNAiso Plus (TaKaRa, Otsu, Shiga, Japan) according to the manufacturer’s instructions. After the total RNA extraction and DNase I treatment, the concentration and quality of each sample was examined using a NANODROP2000 (Thermo Scientific, Wilmington, DE) and Agilent 2100 Bioanalyzer. The mRNA was isolated by magnetic beads with Oligo (dT) and then synthesized to cDNA. Short fragments were purified and resolved with EB buffer for end reparation and single nucleotide A (adenine) addition. After that, the short fragments were connected with adapters. During the quality control (QC) steps, Agilent 2100 Bioanaylzer and ABI StepOnePlus Real-Time PCR System were used in quantification and qualification of the sample libraries. Finally, the libraries were sequenced using Illumina HiSeq™ 2000. Four time points (0, 0.5, 4 and 8 h) were selected for RNA-Seq analysis to obtain a general overview transcriptome of the eggplant peel samples that had been subjected to different light exposures. Three biological replicates were used in the RNA-Seq experiments involving all the four time points, and it means that a total of 12 samples were harvested to construct libraries. Pearson’s correlation was exploited to measure the relevance between each two samples.

### Bioinformatics analysis

The raw reads produced by Illumina HiSeq™ 2000 were subjected to QC which can determine whether a resequencing step is needed or not. After read evaluated and filtered of low QC reads, reads were aligned to the eggplant genome sequences (http://eggplant.kazusa.or.jp/) [58]. And the alignment data was utilized to calculate distribution of reads on reference genes and mapping ratio. After alignment result passing QC, gene and isoform expression analysis was carried out. The differentially expressed genes (DEGs) were selected by a threshold of False Discovery Rate (FDR) ≤ 0.001 and an absolute Log_2_Ratio value ≥1 among the three biological replicates based on the analysis method of the poisson distribution. And the DEGs between groups were screened according to the following criteria: Fold change ≥2 and diverge probability ≥0.8 using Noiseq package method [59]. Further, the sequences of DEGs were compared with the NCBI non-redundant (Nr) database [60], Gene Ontology (GO) [61] and Kyoto Encyclopedia of Genes and Genomes (KEGG) [62] databases to identify and annotate the obtained DEGs using Blast software [63]. In addition, the Log2 (folds of mean RPKM values to the 0 h time point) were used to generate cluster diagrams by MultiExperiment Viewer software with a colour scale (MeV v4.4.1, http://www.tm4.org/) using the hierarchical clustering method [64].

### RNA-seq data validation

qRT-PCR analysis was performed to validate the accuracy of the gene expression obtained from the assembled transcriptome via RNA-Seq. The correlation between the RNA-seq data and qRT-PCR result was analyzed by using SigmaStat 3.0 (SPSS, Chicago, IL). Total RNA were extracted from the peel samples by the MiniBEST Universal RNA Extraction Kit (TaKaRa) and examined using a NANODROP2000 (Thermo Scientific, Wilmington, DE) and characterized on 1.2% agarose gel electrophoresis. And the eligible RNA was selected based on a threshold of absorbance ratio 260/280 ≥ 1.8 and 260/230 ≥ 1.8. 500 ng RNA was synthesized into cDNA with the PrimeScript™ RT Master Mix (Perfect Real Time) (Takara). According to the manufacturer’s instructions of SYBR® Premix Ex Taq™ II (Tli RNaseH Plus) (Takara), qRT-PCR was performed on CFX Real Time PCR Detection System (BioRAD) using the following program: 95 °C for 30 s, followed by 40 cycles of 95 °C for 5 s and 60 °C for 30 s. The Actin gene (*Sme2.5_00072.1_g00003.1*) from eggplant was amplified in parallel as an internal reference gene [65]. The relative expression levels of the amplified products were analyzed using the 2^-ΔΔCT^ method [66]. All qRT-PCR analyses were performed in three biological and three technical replications. The primers employed for this study are listed in Additional file 1.

## Results

### Anthocyanin biosynthesis structural genes were induced by light within an 8 h exposure period

**Fig. 1:**
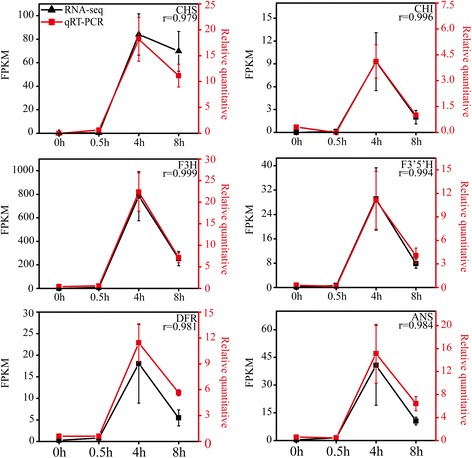
The expression patterns of 6 structural genes and correlation analysis of the RNA-seq data and qRT-PCR result. Correlation analysis showed correlation between RNA-seq data and qRT-PCR was good (Pearson *R* > 0.99). The left y-axis indicates the corresponding expression data of RNA-seq (black lines). The right y-axis shows the relative gene expression levels detected by qRT-PCR (red lines). The x-axis shows the length of time (h) the eggplant peel was exposed to light

The transcriptions of *CRY1*, *CRY2*, *HY5* and *MYB1* were previously shown to be triggered by exposure to light over an 8 h period [5]. Here, the expression levels of six anthocyanin biosynthesis structural genes (*CHS*, *CHI*, *F3H*, *F3’5’H*, *DFR* and *ANS* which were homologous with the genes reported in *Arabidopsis*) were analyzed by qRT-PCR (Fig. 1). The expression patterns of the six structural genes were similar to those of *CRY1*, *CRY2*, *HY5* and *MYB1* [5]. The qRT-PCR analysis showed that the expression levels of most of anthocyanin biosynthesis structural genes were up-regulated slightly at 0.5 h, peaked at 4 h and trended back at 8 h. And similar expression patterns were found in *CRY1* and *HY5* [5]. Thus the four time points were chosen to analyze the transcriptome of ‘*Lanshan Hexian*’ after bags removal using RNA-seq expecting to capture majority of transcriptional changes in anthocyanin biosynthesis related genes.

### Transcriptional response of light by RNA-seq

**Fig. 2:**
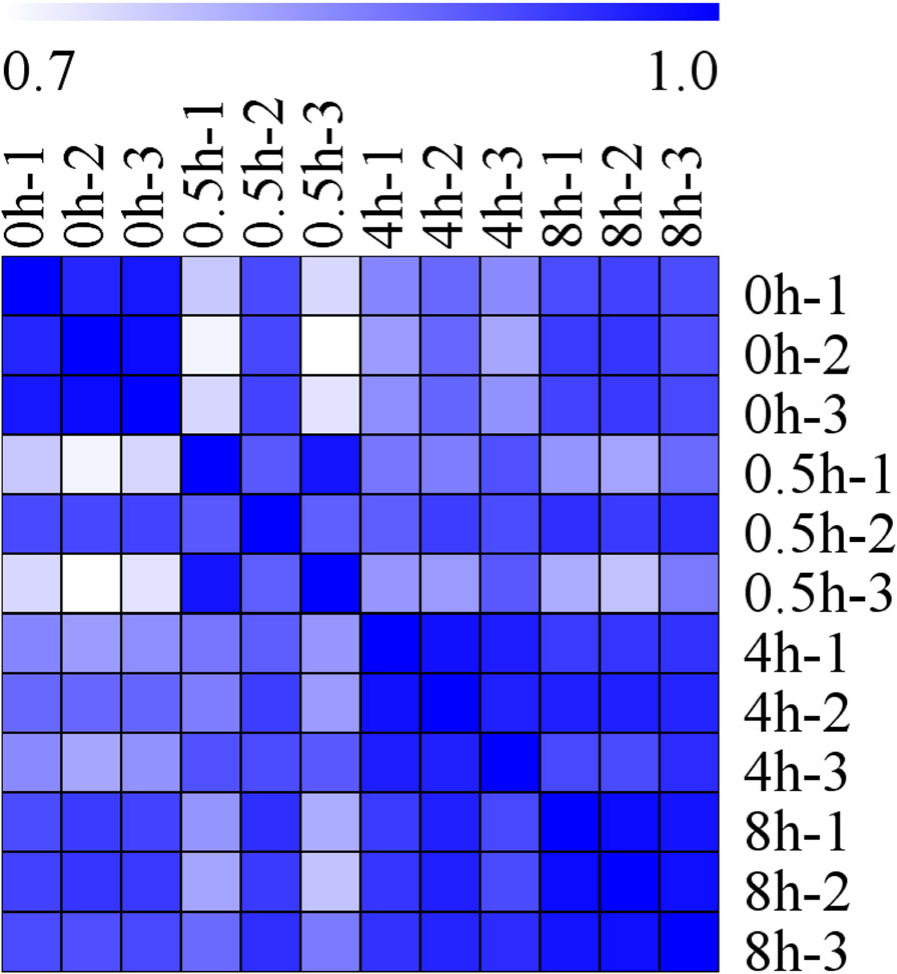
Heat map representation of the correlations coefficient between each two samples. The color represents the correlation coefficient (the darker the color, the higher the correlation, the lighter the color, the lower the correlation)

**Fig. 3:**
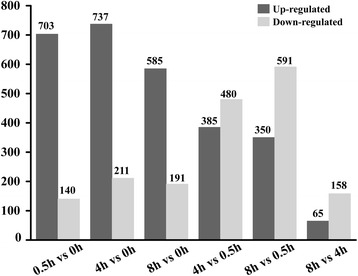
Overview of differentially expressed genes (DEGs)

An average of 49 million clean reads was obtained from each sample, of which 79~ 81% could be mapped to the eggplant genome (http://eggplant.kazusa.or.jp/): 46~ 50% could be mapped to eggplant genes (Additional file 2). Finally, the reads were assembled into a total of 32,629 genes or transcripts (Additional file 3). To test the samples chosen was reliable, the correlation value between each two samples was calculated based on FPKM (Fig. 2 and Additional file 4). A false discovery rate ≤ 0.001, an absolute Log_2_Ratio value ≥1 and diverge probability ≥0.8 were the thresholds applied to assess the significance of differences in gene expression. There were 1956 DEGs detected by means of time point to time point comparisons, including 843 (0.5 h vs 0 h), 948 (4 h vs 0 h), 776 (8 h vs 0 h), 865 (4 h vs 0.5 h), 941 (8 h vs 0.5 h) and 223 (8 h vs 4 h) (Fig. 3 and Additional file 5). All the known anthocyanin biosynthesis related genes were involved in the DEGs. The RNA-Seq and qRT-PCR results for these genes were highly correlated (*r* = 0.97~ 1.000) (Fig. 1). In addition, 10 selected genes, which had different expression patterns after bag removal, were chosen to validate the RNA-Seq results by qRT-PCR (*r* = 0.91~ 1.000) (Additional file 6). These results provided further validation of the reliability of the transcriptome data.

### GO analysis of the DEGs

**Fig. 4:**
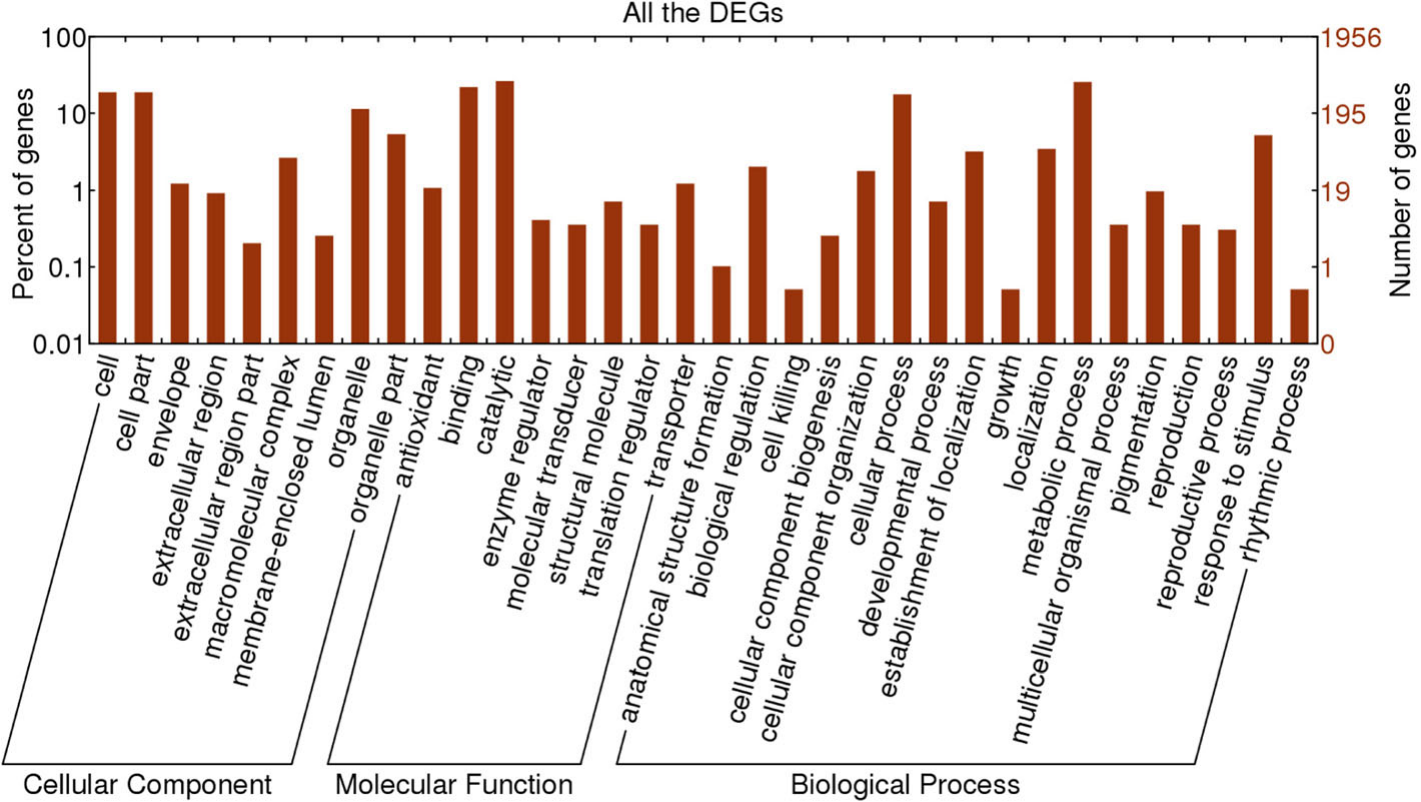
GO categories assigned to DEGs. The left y-axis shows the percentages of proteins identified, and the right y-axis shows the protein number. The proteins were categorized according to the annotation of GO, and the number of each category is displayed based on biological process, cellular components, and molecular functions

The GO consortium provides a standard vocabulary that is dynamic and controlled, and that can be applied to all eukaryotes even though knowledge of the roles of genes and cell proteins is accumulating and expanding [67]. We therefore used GO assignments to classify the functions of the DEGs in eggplant peel. Of the total of 1956 DEGs, 873 have been annotated according to the GO database and are classified as ‘biological process’, ‘cellular component’ and ‘molecular function’. The results covered 34 important functional groups including 17 for biological process, 9 for cellular components, and 8 for molecular function (Fig. 4). The two largest subcategories in the ‘biological process’ category were ‘metabolic process’ including 500 DEGs and ‘cellular process’ including 344 DEGs. In the ‘cellular component’ category, the two largest subcategories were ‘cell’ and ‘cell part’ and both included 367 DEGs. For the ‘molecular function’ category, ‘catalytic activity’ including 514 DEGs and ‘binding activity’ including 429 DEGs were the most abundant.

### Cluster analysis of expression patterns in the DEGs

**Fig. 5:**
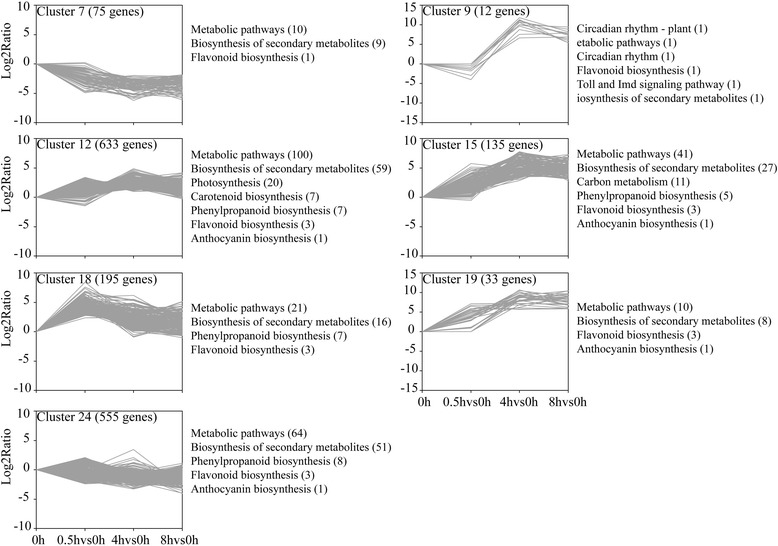
Cluster analysis of DEGs with significant expression profile changes and KEGG pathway enrichment analysis. All the DEGs were subjected to complete-linkage hierarchical clustering using a Euclidean distance metric and divided into 26 clusters. The x-axis showed the time point to time point comparison. The y-axis shows the relative log2(ratio) of each comparison. Enriched KEGG pathways are listed to the right of each cluster

The 1956 DEGs were subjected to complete-linkage hierarchical clustering using a Euclidean distance metric by MeV v4.4.1 software and divided into 26 clusters (Additional file 7). We also present an overview of the KEGG pathway enrichment to provide a global description of the enriched biological pathways in each cluster of similarly regulated transcripts. The structural genes in anthocyanin biosynthesis pathway were divided into three pathways, including ‘phenylpropanoid biosynthesis’, ‘flavonoid biosynthesis,’ and ‘anthocyanin biosynthesis’. These pathways were enriched in clusters 7, 9, 12, 15, 18, 19 and 24 (Fig. 5). Therefore, genes in these clusters were the focus of further attention in this study. In addition, ‘metabolic pathways’ and ‘biosynthesis of secondary metabolites’ were highly enriched in six out of seven clusters.

As shown in Fig. 5, the expression level of genes in cluster 7 declined during this period. The expression level of genes in cluster 9 were down-regulated at 0.5 h but up-regulated to peak level in the samples taken after 4 h exposure to light, and maintained a high expression level. The genes in cluster 12, 15 and 19 were up-regulated slightly at 0.5 h, peaked after 4 h exposure to light, and maintained a high expression level at 8 h. Cluster 18 and 24 contained genes that were up-regulated to peak level in the samples taken after 0.5 h, but then the expression level of genes decreased after 4 and 8 h (Fig. 5). From the above, we found the genes in cluster 12, 15 and 19 have similar expression pattern but with different relative Log_2_ratio value at the same time point after eggplants exposure to light. Therefore, the genes in cluster 12, 15 and 19 could be analyzed together.

### The structural anthocyanin biosynthesis genes

**Fig. 6:**
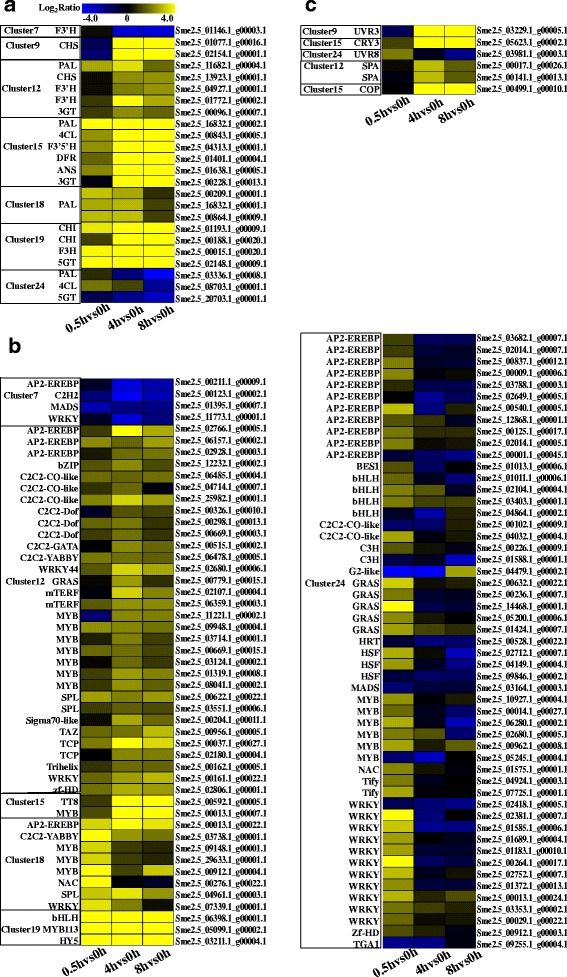
Heat map representation of the genes expression patterns. **a** The structural anthocyanin biosynthesis genes, **b** All the transcription factors response to light, **c** The photoreceptors and genes related to light signal transduction. The color scale represents the Log2 (fold-change to the 0 h time point). Genes that are upregulated appear in yellow, and those that are downregulated appear in blue, with the relative Log2Ratio reflected by the intensity of the color

Twenty-four structural anthocyanin biosynthesis genes were identified. They participated in each step of anthocyanin metabolic pathway. 2 *PAL*, *4CL*, *CHS*, 2 *CHI*s, *F3H*, 2 *F3’H*s, *F3’5’H*, *DFR*, *ANS*, 2 *3GT* and *5GT* presented in cluster 12, 15 and 19. The expression levels of these genes were increased and peaked after 4 h. In cluster 18, another three *PAL* genes were identified which expression level with peaked expression level at 0.5 h time point. The expression level of the exception, *Sme2.5_03336.1_g00008.1* (*PAL*), another *4CL* and *5GT* were specifically induced after 0.5 h light exposure but then their activity declined. Another two *CHS* genes were down-regulated after 0.5 h, but then up-regulated were divided into cluster 9. The transcriptional level of another *F3’H* gene was down-regulated over the period after exposure to light (Fig. 6a).

### Transcription factors

One hundred and thirty-eight transcription factors that exhibited highly dynamic changes in response to light were found, 102 of which were present in the 7 clusters. Among the 102 transcription factors, *MYB* (18 genes) constituted the largest group, followed by *AP2-EREBP* (16 genes), *WRKY* (15 genes), *GRAS* (6 genes), *bHLH* (6 genes), *C2C2-CO-like* (5 genes), *C2C2-Dof* (3 genes), *HSF* (3 genes), *SPL* (3 genes), *zf-HD* (2 genes), *MADS* (2 genes), *mTERF* (2 genes), *TCP* (2 genes), *NAC* (2 genes), *C3H* (2 genes), *Tify* (2 genes), *C2C2-YABBY* (2 genes), *G2-like*, *Sigma70-like*, *C2C2-GATA*, *C2H2*, *Trihelix*, *HRT*, *HY5*, *bZIP*, *TAZ*, *TGA1*and *BES1* (Fig. 6b). Strikingly, *HY5*, *MYB113*, *TT8* and *WRKY44* (*TTG2*), which were known as anthocyanin biosynthesis regulated genes [25, 31, 46, 68, 69], were divided into clusters 12, 15 and 19 (Fig. 6b). In the three clusters, another 8 *MYB*s, 3 *C2C2-CO-like*s, 2 *SPL*s, *bZIP*, *WRKY* and *bHLH* were found. Previous studies have reported that genes which belong to the same gene families with these transcription factors participated in anthocyanin biosynthesis [24, 28, 31, 69–73]. Thus, we suggested that these transcription factors might be involved in light-induced anthocyanin biosynthesis.

### Plant light signal perception and transduction

Under light condition, photoreceptors act as central promotors to substitute ubiquitination and degradation of the positive regulators in anthocyanin biosynthesis pathway caused by COP1, such as HY5 [42] and MYB1 [46, 47]. Here, *UVR3*, *CRY3* and *UVR8* were identified in clusters 9, 15 and 24, respectively (Fig. 6c). The expression levels of *CRY3* and *UVR3* were changed slightly at 0.5 h time point, increased to the highest level at 4 h time point but then declined slightly at 8 h time point. By contrast, the expression level of *UVR8* was increased to a peak after 0.5 h exposure to light, declined towards the control level after 4 h and continued to decrease for the next 4 h. The COP1/SPA complex act as a molecular switch of light-induced anthocyanin biosynthesis located downstream of the photoreceptors. In this study, *COP1* and two *SPA*s were found in clusters 12 and 15 (Fig. 6c). KEGG pathway analysis showed that the three genes were enriched in ‘ubiquitin mediated proteolysis’.

## Discussion

In our previous study, anthocyanins couldn’t accumulate in eggplant peel under shaded conditions. After eggplants were exposed to light, anthocyanins rapidly synthesized within a short time [5, 35]. These findings indicated that the coloration of eggplants peel completely depend on light, as observed in previous studies on lithci, Chinese bayberry, grape berry, apple, and red pear [6, 36, 47, 57]. The existing studies focused on exploring the molecular regulatory mechanisms associated with the accumulation of anthocyanins, including the paper we recently published [6, 7, 35]. However, whether and how does light participate in regulating anthocyanin biosynthesis before color becomes evident have not been studied so far. Here, RNA-seq was used to explore the relationship between light and the expression of candidate genes, which might be involved in anthocyanin biosynthesis over an 8 h period in eggplant.

Jiang et al. has reported that the transcriptional levels of *CRY1*, *CRY2*, *HY5* and *MYB1* were significantly induced by exposure to light over an 8 h period [5]. In addition, qRT-PCR analysis showed that the expression levels of six anthocyanin biosynthesis structural genes were also affected in our pre-preparation work. Then another 18 structural anthocyanin biosynthesis genes were identified as DEGs by RNA-seq analysis and divided into 7 clusters. Most of them were up-regulated after exposure to light for 4 h. These results suggested that the light response of these genes is dependent upon some upstream transcription factors. For instance, HY5 is necessary for the rapid transcription of *F3H* and *CHS* during the dark-to-light transition, which eventually allows the accumulation of anthocyanins [74]. In addition, the expression patterns of these anthocyanin biosynthesis structural genes corresponded to the light intensity fluctuation which was parabolic in the day. This result might reveal the direct reason why eggplant peel shows poor color production under weak light conditions.

In this study, 102 transcription factors which were significantly regulated by light were involved in the 7 clusters. Among these transcription factors, the well-known anthocyanin biosynthesis regulating genes including *HY5* [31], *MYB113* [31], *TT8* [9] and *WRKY44* (*TTG2*) [69] were found. As is well known, HY5 [42, 51] and MYB [46] were repressed by the activity of COP1 in darkness, but they triggered expression of light-inducible genes, such as the gene encoding CHS after light exposure [75]. In addition, *MYB1* [76, 77], *MYB2* [12], *MYB5* [70], *COL11* (*C2C2-CO-like*) [28], *MADS* [78], *MYC2* (*bHLH*) [79] and *SPL* [29] were reported to participate in anthocyanin biosynthesis in various plant species. In addition, the connections between other transcription factors and anthocyanin synthesis have attracted much attention. For example, the family genes of *WRKY* [69], *C2C2*-*CO*-*like* [28, 80] and *NAC* [81] were also reported to be involved in anthocyanin biosynthesis. In the present study, 15 *WRKY*s, 6 *GRAS*s, 5 *C2C2*-*CO*-*like*s, 3 *SPL*s and *NAC* were screened in the seven clusters that included anthocyanin biosynthesis structural genes. These genes might be involved in the regulation of anthocyanin biosynthesis. Also, these genes point to the direction for further studies of anthocyanin biosynthesis in eggplants.

COP1 is an E3 ubiquitin ligase that represses photomorphogenesis by mediating light-dependent degradation of various transcription factors involved in light signaling. The subcellular localization of COP1 is light dependent [82]. In darkness, COP1 is situated in the nucleus where it interacts with photomorphogenesis-promoting transcription factors. When plants are exposed to light, the concentration of COP1 in the nucleus declines rapidly [50]. *SmCOP1*, which is annotated as *Sme2.5_00128.1_g00013.1*, is functionally complemented in phenotypes of corresponding *Arabidopsis* mutants where it interacts with SmHY5 and SmMYB1 (SmMYB113) [5]. In this study, another *COP1* annotated as *Sme2.5_00499.1_g00010.1,* and two *SPA* genes were located in clusters 12 and 15. Their expression levels kept control level at 0.5 h time point, increased to the highest level at 4 h time point but then declined a little at 8 h time point. Strikingly, the expression level of *Sme2.5_00128.1_g00013.1* showed little change after eggplants were exposed to light when compared to control level. Stacey et al. [82] reported that light is the control of COP1 partitioning between nucleus and cytoplasm. Maier and Hoecker [83] found that mutations in the *COP1/SPA* complex, which is the most important repressor of light signaling, cause a hyperaccumulation of anthocyanins in normal and high intensity light. Taking into account the previous information from *Arabidopsis*, we concluded that the expression level of *COP1* may be not be regulated by light and the function of the COP1 protein was to combine the photoreceptors but not the positive regulators, in the context of anthocyanin biosynthesis. These results suggest that *Sme2.5_00499.1_g00010.1* may participate in light-induced anthocyanin biosynthesis in a different way.

Three photoreceptors in eggplant, *CRY3*, *UVR3*, and *UVR8*, were identified as DEGs in this study. Jiang et al. [5] suggested that a blue-light-triggered CRY1/CRY2-COP1 interaction allows HY5 and MYB1 to combine with the downstream anthocyanin biosynthesis genes (CHS and DFR) in eggplant. These results from this later experiment suggest that the expression of *CRY3* was stimulated by light, but *CRY1* and *CRY2* were not thus stimulated. Also, the expression pattern of *CRY3* was similar to those of *CRY1* and *CRY2* [5]. The anthocyanin content and the expression level of *UVR8* were also elevated under UV-B in peach, strawberry, lettuce and radish sprouts [84–87]. In this study, *UVR8* was up-regulated by light after 0.5 h exposure and was down-regulated after 4 and 8 h. It is possible that unknown genes negatively regulate the expression of *UVR8* under sunlight. In addition, *UVR3* encodes a photolyase that is specific for 6,4 photoproducts in *Arabidopsis thaliana* but which is not activated by light [88]. In eggplant peel the *UVR3* mRNA level was increased within 4 h after bags removal suggesting that *UVR3* responds to light differently in eggplant.

Comparing the transcriptome data with our previous studies [35], we found that the 1956 DEGs could be divided into two parts: 869 genes could be identified as DEGs in both the two studies (Additional file 8) and the other 1087 genes were only identified as DEGs in this study. Then the biological processes and pathways involved in the 869 DEGs were identified by GO and KEGG pathway enrichment analysis (Additional files 9 and 10). Except for the ‘anatomical structure formation’ in the ‘biological process’ category was not found, the functional groups involved in the 869 DEGs were same with which involved in all the DEGs (Additional file 9 and Fig. 4). KEGG pathway enrichment analysis showed that ‘phenylpropanoid biosynthesis’, ‘flavonoid biosynthesis,’ and ‘anthocyanin biosynthesis’ pathways were enriched in the 869 DEGs (Additional file 10). Further analysis found that 22 structural genes, 37 transcription factors and 3 photoreceptors were included in the 869 DEGs. Strikingly, 3 *AP2-EREBP*s, 3 *WRKYs*, 2 *bHLH*s, 2 *MYB*s, 2 *C2C2*-*CO*-*like*s, *C2C2*-*YABBY*, *GRAS*, *HSF*, *MADS*, *mTERF*, *SPL*, *TAZ* and *TGA1* were found in the 37 transcription factors except for *HY5*, *MYB113*, *TT8* and *WRKY44*. These results suggested that these 869 DEGs participated in the light-induced anthocyanin biosynthesis. As for the identification of the other 1087 DEGs, this might be consequence of the light condition change, the circadian clock or other factors.

## Conclusion

In this study, RNA-seq was applied to elucidate the underlying molecular mechanism of light-induced anthocyanin biosynthesis in eggplant peel before color becomes evident. A total of 1956 DEGs were identified and divided into 26 clusters based on the distinct expression patterns. By comparing the transcriptome data with our previous study [35], the structural genes, photoreceptors, light signal transduction elements and transcription factors involved in the anthocyanin biosynthesis pathway could be found in the intersection of the two transcriptome data. These results suggested that light induce anthocyanin biosynthesis from the moment of exposure to light to the saturation of anthocyanin. This study will expand our understanding of the mechanism of light-induced anthocyanin biosynthesis in plant, and point to new directions in the study of anthocyanin biosynthesis.

## Additional files

Additional file 1: **Table S1.** List of primers sequences used in this study. (DOCX 33 kb)

Additional file 2: **Table S2.** Summary statistics of sequencing and assembly. (DOCX 28 kb)

Additional file 3: **Table S3.** All the assembled genes in this project. Gene_id: gene ID number; FPKM: FPKM value of this gene; the differentially expressed genes (DEGs) were annotated by overstriking. (XLS 5248 kb)

Additional file 4: **Table S4.** Correlation value between each two samples. (XLS 25 kb)

Additional file 5; **Table S5.** All the identified DEGs in this study. Gene_id: gene ID number; TAIR10: gene ID in Arabidosis corresponding to the degs in eggplant; E-value: The lowest E-value of Blast-nr for each genes; FPKM: FPKM value of this gene; Means-0 h:mean expression (FPKM) of 0 h group; log2Ratio(s2/s1): Log2(folds of mean expression in two groups); Probability:Probability of difference; Blast nr: NR annotation. (XLS 1400 kb)

Additional file 6: **Figure S1.** Correlation analysis of the RNA-seq data and qRT-PCR result. Correlation analysis showed correlation between RNA-seq data and qRT-PCR was good (Pearson *R* > 0.9). The left y-axis indicates the corresponding expression data of RNA-seq (black lines). The right y-axis shows the relative gene expression levels detected by qRT-PCR (red lines). The x-axis shows the length of time (h) the eggplant peel was exposed to light. (DOCX 1112 kb)

Additional file 7: **Figure S2.** Cluster analysis of DEGs with significant expression profile changes. All the DEGs were subjected to complete-linkage hierarchical clustering using a Euclidean distance metric and divided into 26 clusters. The x-axis showed the time point to time point comparison. The y-axis shows the relative log2(ratio) of each comparison. (DOCX 1568 kb)

Additional file 8: **Table S6** The 869 DEGs identified in both this study and the previous study [35]. (XLS 636 kb)

Additional file 9: **Figure S3.** GO categories assigned to the 869 DEGs. The left y-axis shows the percentages of proteins identified, and the right y-axis shows the protein number. The proteins were categorized according to the annotation of GO, and the number of each category is displayed based on biological process, cellular components, and molecular functions. (DOCX 798 kb)

Additional file 10: **Table S7** KEGG pathway enrichment analysis of the 869 DEGs. (DOCX 40 kb)

## Abbreviations

4CL: 4-coumarate-CoA ligase
ANS: Anthocyanin synthase
AT: Acylation
CHI: Chalcone isomerase
CHS: Chalcone synthase
COL: CONSTANS-like
COP1: Constitutive Photomorphogenic 1
CRY3: Cryptochrome 3
DEGs: Differentially expressed genes
DFR: Dihydroflavonol reductase
F3H: Flavanone 3-hydroxyl enzyme
FDR: False discovery rate
GO: Gene ontology
GT: Glycosylation
HFR: Hypocotyl in Far-Red 1
HY5: Hypocotyl 5
HYH: Hypocotyl homolog
KEGG: Kyoto encyclopedia of genes and genomes
LAF1: Long After Far-Red Light 1
LDOX: Leucoanthocyanidin dioxygenase
LZF: Light-regulated zinc finger protein
MT: Methylation
Nr: NCBI non-redundant database
PAL: Phenylalanine ammonia lyase
PHOTs: Phototropins
PHYs: Phytochromes
QC: Quality control
SPL9: Squamosa promoter binding protein-like 9
SRA: Sequence read archive
UVR8: UV Resistance Locus 8
