# Correction to: Transcriptome profiling of genes related to light-induced anthocyanin biosynthesis in eggplant (*Solanum melongena* L.) before purple color becomes evident

**DOI:** 10.1186/s12864-018-4693-y

**Published:** 2018-05-04

**Authors:** Jing Li, Yong-Jun He, Lu Zhou, Yang Liu, Mingmin Jiang, Li Ren, Huoying Chen

**Affiliations:** 1 School of Agriculture and Biology, Shanghai Jiao Tong University, 800 Dongchuan Road, Minhang District, Shanghai, 200240 China; 2 Institute for Agri-Food Standards and Testing Technology, Shanghai Academy of Agricultural Sciences, 1000 Jinqi Road, Fengxian District, Shanghai, 201403 China

## Correction

After publication of the original article [1] it was noted that in Additional file 1: Table S1, and Fig. 1, specific primer sequences were incorrect, and taken from Sme2.5_02154.1_g00001.1 rather than Sme2.5_13923.1_g00001.1.

The primer sequences of *SmCHS* refer to the author’s previous study entitled as “Molecular cloning and characterization of anthocyanin biosynthesis genes in eggplant (*Solanum melongena* L.)” [2], which has been published on Acta Physiologiae Plantarum. In that study, *SmCHS* was not annotated with the code name from Eggplant Genome Database. However, in the original paper which this Correction refers to, the protein sequence of AtCHS (AT5G13930.1) was employed as a query to search against the Eggplant Genome Database and the results were showed as following:

**Table:** 

Sequences producing significant alignments:	Score (bits)	E-Value
Sme2.5_02154.1_g00001.1	661	0.0
Sme2.5_13923.1_g00001.1	656	0.0

Therefore, the authors had interpreted that SmCHS was Sme2.5_02154.1_g00001.1 in this study.

The correct Additional file 1 and Fig. 1 are included with this manuscript.
