# Supplementary material for: Transcriptome profiling of genes related to light-induced anthocyanin biosynthesis in eggplant (Solanum melongena L.) before purple color becomes evident
Source: BMC Genomics. 2018 Mar 20;19:201. doi: 10.1186/s12864-018-4587-z (PMC5859761; doi:10.1186/s12864-018-4587-z)
Supplement: Supplementary file 1 — Table S1. List of primers sequences used in this study. (DOCX 33 kb) [file 12864_2018_4587_MOESM1_ESM.docx]

**Additional file 1: Table S1. List of primers sequences used in this study**

| Gene code | Gene name | Forward primer (5’-3’) | Reverse primer (5’-3’) |
| --- | --- | --- | --- |
| Sme2.5_02154.1_g00001.1 | CHS | GGGAACAGTACTCCGGCTAGCC | AACACCTGAAATTGGGTCTGAACCA |
| Sme2.5_01193.1_g00009.1 | CHI | CCTTGACGGGTAAGCAATACTCTG | GATGGAGGCACCATGTGGGAAGG |
| Sme2.5_00015.1_g00020.1 | F3H | GTGGTCCAAGACTGGCGTGAAAT | TTCTCTAACCCCATTGCTTCTGATA |
| Sme2.5_04313.1_g00001.1 | F3’5’H | TGGACCTCGTTGGAAGTTGCTAAG | TGCCATCGCGAACGTCAACATAT |
| Sme2.5_01401.1_g00004.1 | DFR | GGCCATTGAGACTTGCCGACAG | CACCATTGGTCAACTGTCCTGTACT |
| Sme2.5_01638.1_g00005.1 | ANS | CTCGATTCCCACCTCGGACCTT | TCAGCTGCAGCGTCCTGTTTGT |
| Sme2.5_01116.1_g00007.1 | SKS4^1^ | GGCACAACATGTCCAATTCCACCT | CTGGAATTCGAGGACGGCTATAG |
| Sme2.5_06398.1_g00001.1 | ICE1^2^ | GGCCAATCTTCCACCACTAAGGAAT | GACAGTGCTGGCTGCATTGGAGCT |
| Sme2.5_10149.1_g00003.1 | MYC2-like^3^ | TGGGTTCCGCTGATGTTATCCAAGA | CCGTTATTAACATTTCCACGTTACT |
| Sme2.5_04997.1_g00006.1 | GSTU8^4^ | GCTTTAGCGCGTTTCTGGGCTACAT | CCAAATTTATCACCAACAAAGAACT |
| Sme2.5_00145.1_g00010.1 | GA2OX2^5^ | CGGTTGGGTTGAATACATTCTCGT | CCAGAATCTCACACGACATCTTCTT |
| Sme2.5_09148.1_g00001.1 | MYB77^6^ | AAGGGTAGGTCCGGCAAATCCTG | CATTATCGGTCCGACCCGGAAGTA |
| Sme2.5_03009.1_g00002.1 | E3 ubiquitin-protein ligase RING1-like | GTGTTTCACGCCTTCCGATGATCAC | GAGAGCCATTTGGTAAGGCAATTTG |
| Sme2.5_03694.1_g00004.1 | PGD^7^ | GGAATGGGAGTGTCAGGTGGCGA | CCAGAACCTCCTTCACCAATATATG |
| Sme2.5_00499.1_g00004.1 | GAD^8^ | GAGCTTCAGAATCGATGTGTGAAC | GCTTGTCATAGGGCTTTCCTTGG |
| Sme2.5_03164.1_g00002.1 | MADS^9^ | GAAGCTTAAAGCAAGAGTGGAAGT | CAGAAAGTTGATCAAACATGTGTTGT |
| *Smactin* |  | GTCGGAATGGGACAGAAGGATG | GTGCCTCAGTCAGGAGAACAGGGT |

1: SKS4: L-ascorbate oxidase homolog

2: ICE1: transcription factor ICE1-like

3: MYC2-like: transcription factor MYC2-like

4: GSTU8: glutathione S-transferase TAU 8

5: GA2OX2: gibberellin 2-oxidase

6: MYB77: myb domain protein 77

7: PGD: 6-phosphogluconate dehydrogenase

8: GAD: glutamate decarboxylase

9: MADS: MADS-box transcription factor
