# Supplementary material for: Transcriptome profiling of genes related to light-induced anthocyanin biosynthesis in eggplant (Solanum melongena L.) before purple color becomes evident
Source: BMC Genomics. 2018 Mar 20;19:201. doi: 10.1186/s12864-018-4587-z (PMC5859761; doi:10.1186/s12864-018-4587-z)
Supplement: Supplementary file 2 — Table S2. Summary statistics of sequencing and assembly. (DOCX 28 kb) [file 12864_2018_4587_MOESM2_ESM.docx]

| Sample Name | Clean reads | Genome map Rate | Gene map Rate | Expressed Gene |
| --- | --- | --- | --- | --- |
| 0.5h-1 | 48936416 | 80.68% | 48.91% | 24169 |
| 0.5h-2 | 49089670 | 80.38% | 49.06% | 24191 |
| 0.5h-3 | 49036646 | 79.55% | 49.15% | 23803 |
| 0h-1 | 49102418 | 80.09% | 49.90% | 23772 |
| 0h-2 | 49103778 | 79.90% | 49.05% | 23371 |
| 0h-3 | 49118046 | 80.25% | 49.07% | 23701 |
| 4h-1 | 48895100 | 79.34% | 48.51% | 23315 |
| 4h-2 | 49095672 | 79.70% | 46.25% | 22623 |
| 4h-3 | 49068724 | 79.65% | 48.87% | 23686 |
| 8h-1 | 49112368 | 79.76% | 47.75% | 23685 |
| 8h-2 | 49129178 | 79.15% | 47.99% | 23683 |
| 8h-3 | 49115242 | 79.38% | 49.28% | 23819 |

**Additional file 2: Table S2.** **Summary statistics of sequencing and assembly**
