# Supplementary material for: Transcriptome profiling of genes related to light-induced anthocyanin biosynthesis in eggplant (Solanum melongena L.) before purple color becomes evident
Source: BMC Genomics. 2018 Mar 20;19:201. doi: 10.1186/s12864-018-4587-z (PMC5859761; doi:10.1186/s12864-018-4587-z)
Supplement: Supplementary file 6 — Figure S1. Correlation analysis of the RNA-seq data and qRT-PCR result. Correlation analysis showed correlation between RNA-seq data and qRT-PCR was good (Pearson R > 0.9). The left y-axis indicates the corresponding expression data of RNA-seq (black lines). The right y-axis shows the relative gene expression levels detected by qRT-PCR (red lines). The x-axis shows the length of time (h) the eggplant peel was exposed to light. (DOCX 1112 kb) [file 12864_2018_4587_MOESM6_ESM.docx]

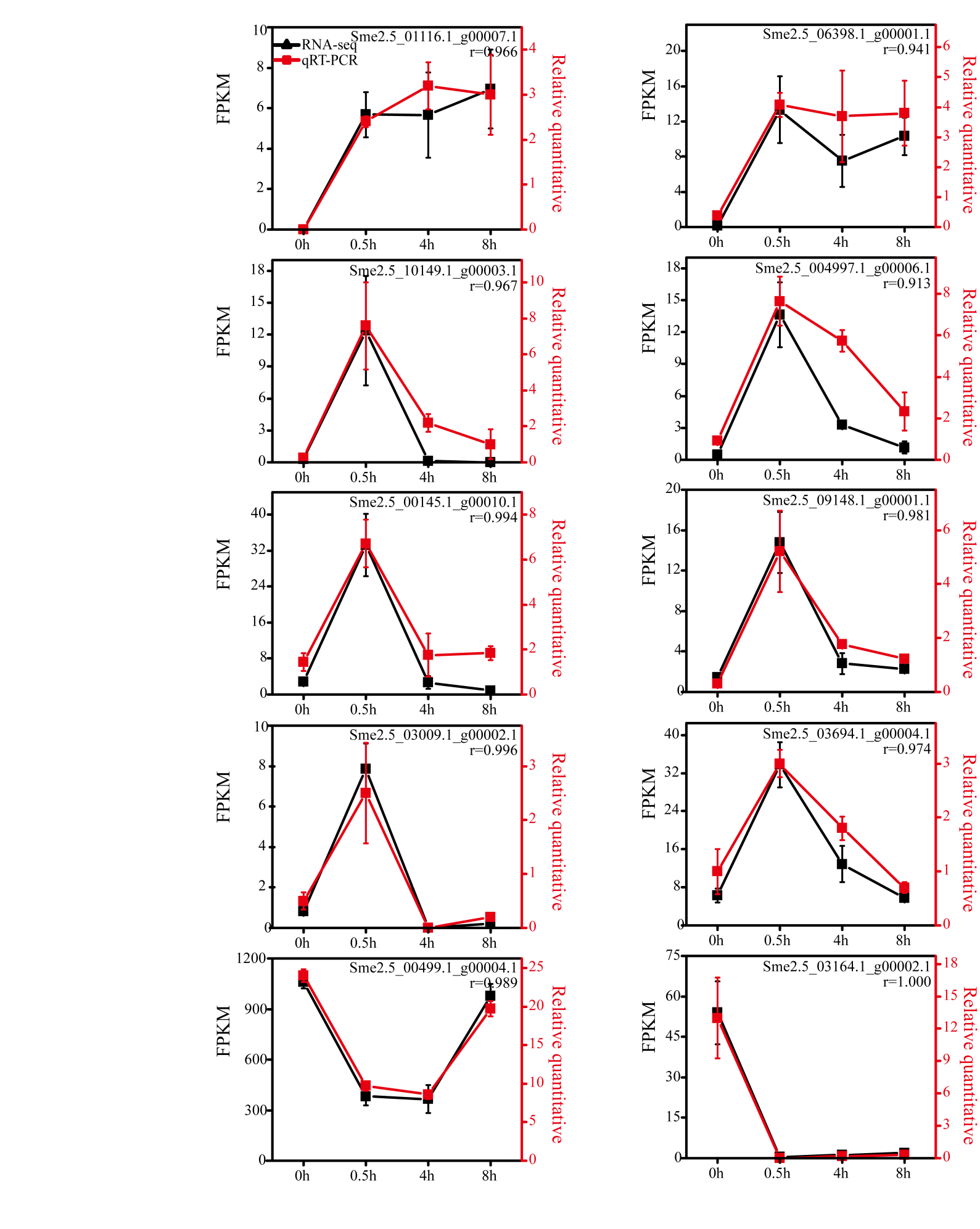


**Additional file 6: Figure S1 Correlation analysis of the RNA-seq data and qRT-PCR result.** Correlation analysis showed correlation between RNA-seq data and qRT-PCR was good (Pearson R > 0.9). The left y-axis indicates the corresponding expression data of RNA-seq (black lines).The right y-axis shows the relative gene expression levels detected by qRT-PCR (red lines). The x-axis shows the length of time (h) the eggplant peel was exposed to light.
