# Supplementary material for: Transcriptome profiling of genes related to light-induced anthocyanin biosynthesis in eggplant (Solanum melongena L.) before purple color becomes evident
Source: BMC Genomics. 2018 Mar 20;19:201. doi: 10.1186/s12864-018-4587-z (PMC5859761; doi:10.1186/s12864-018-4587-z)
Supplement: Supplementary file 7 — Figure S2. Cluster analysis of DEGs with significant expression profile changes. All the DEGs were subjected to complete-linkage hierarchical clustering using a Euclidean distance metric and divided into 26 clusters. The x-axis showed the time point to time point comparison. The y-axis shows the relative log2(ratio) of each comparison. (DOCX 1568 kb) [file 12864_2018_4587_MOESM7_ESM.docx]

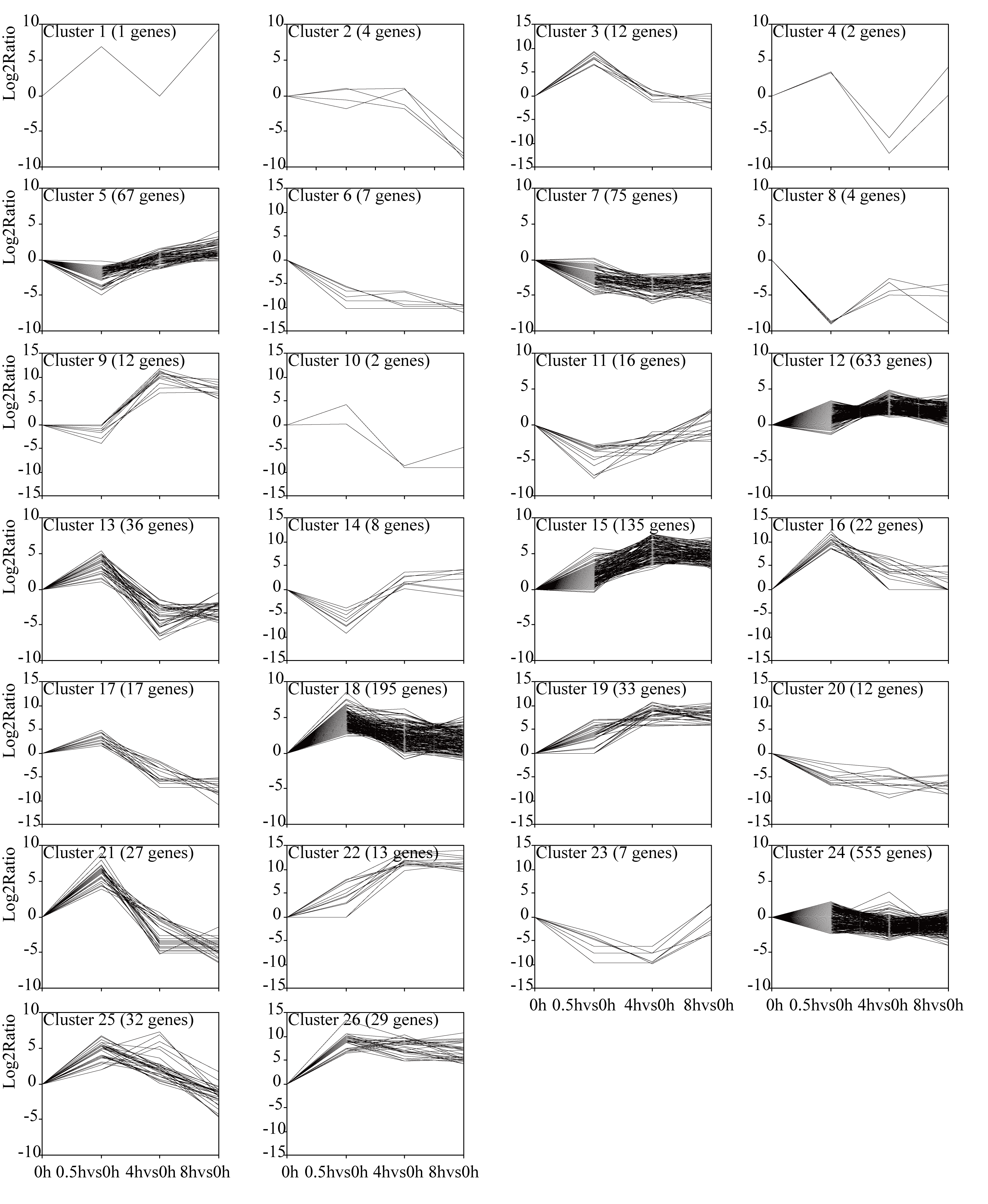


**Additional file 7: Figure S2. Cluster analysis of DEGs with significant expression profile changes.** All the DEGs were subjected to complete-linkage hierarchical clustering using a Euclidean distance metric and divided into 26 clusters. The x-axis showed the time point to time point comparison. The y-axis shows the relative log2(ratio) of each comparison.
