# Supplementary material for: Transcriptome profiling of genes related to light-induced anthocyanin biosynthesis in eggplant (Solanum melongena L.) before purple color becomes evident
Source: BMC Genomics. 2018 Mar 20;19:201. doi: 10.1186/s12864-018-4587-z (PMC5859761; doi:10.1186/s12864-018-4587-z)
Supplement: Supplementary file 9 — Figure S3. GO categories assigned to the 869 DEGs. The left y-axis shows the percentages of proteins identified, and the right y-axis shows the protein number. The proteins were categorized according to the annotation of GO, and the number of each category is displayed based on biological process, cellular components, and molecular functions. (DOCX 798 kb) [file 12864_2018_4587_MOESM9_ESM.docx]

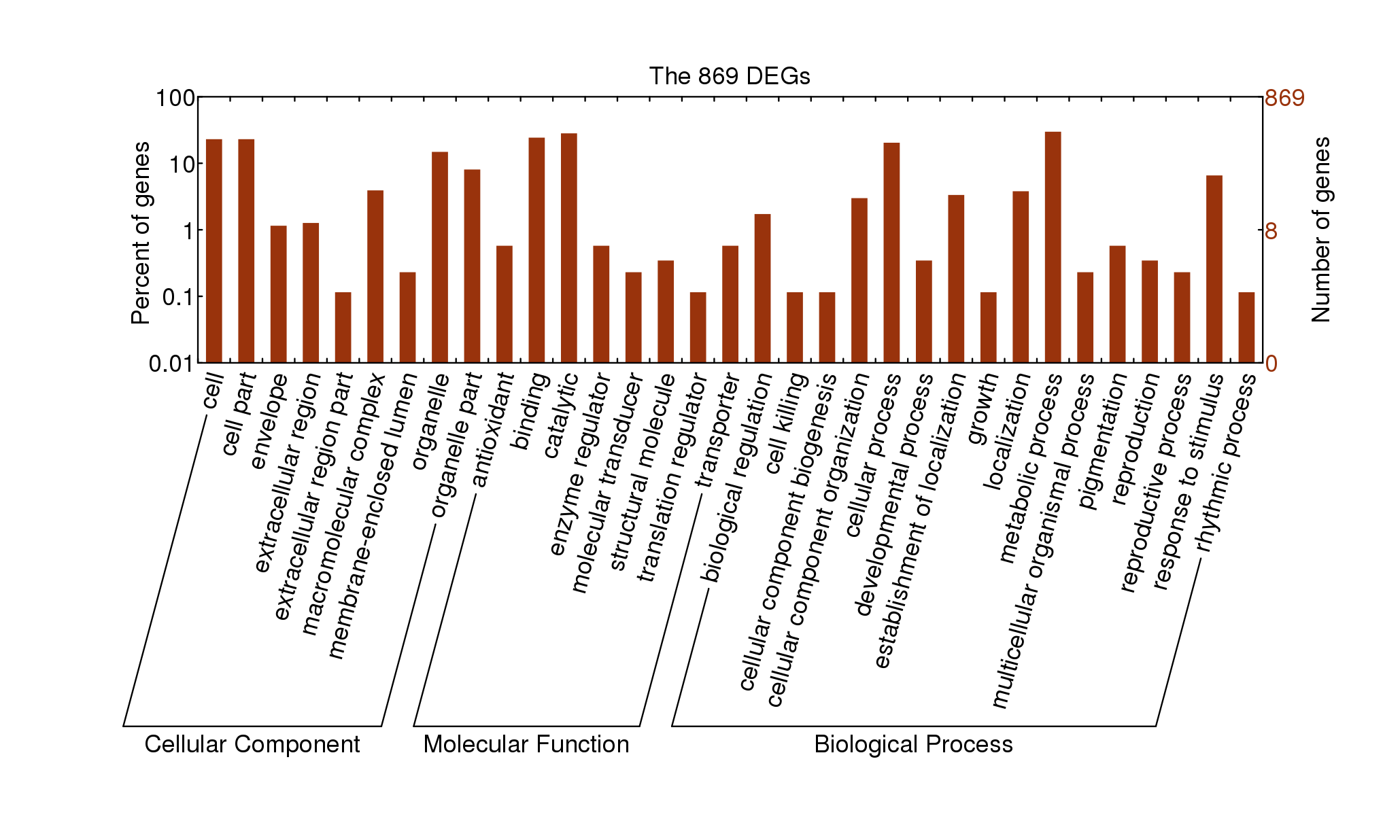


**Additional file 9: Figure S3 GO categories assigned to the 869 DEGs.** The left *y*-axis shows the percentages of proteins identified, and the right y-axis shows the protein number. The proteins were categorized according to the annotation of GO, and the number of each category is displayed based on biological process, cellular components, and molecular functions.
