# Supplementary material for: Transcriptome profiling of genes related to light-induced anthocyanin biosynthesis in eggplant (Solanum melongena L.) before purple color becomes evident
Source: BMC Genomics. 2018 Mar 20;19:201. doi: 10.1186/s12864-018-4587-z (PMC5859761; doi:10.1186/s12864-018-4587-z)
Supplement: Supplementary file 10 — Table S7 KEGG pathway enrichment analysis of the 869 DEGs. (DOCX 40 kb) [file 12864_2018_4587_MOESM10_ESM.docx]

**Additional file 10: Table S7** KEGG pathway enrichment analysis of the 869 DEGs.

| Pathways | DEPs with pathway annotation | Pathway ID |
| --- | --- | --- |
| Metabolic pathways | 123 | ko01100 |
| Biosynthesis of secondary metabolites | 80 | ko01110 |
| Photosynthesis | 24 | ko00195 |
| Biosynthesis of antibiotics | 23 | ko01130 |
| Microbial metabolism in diverse environments | 22 | ko01120 |
| Carbon metabolism | 16 | ko01200 |
| Plant hormone signal transduction | 15 | ko04075 |
| Biosynthesis of amino acids | 14 | ko01230 |
| Protein processing in endoplasmic reticulum | 13 | ko04141 |
| Phenylpropanoid biosynthesis | 12 | ko00940 |
| Plant-pathogen interaction | 11 | ko04626 |
| Photosynthesis - antenna proteins | 11 | ko00196 |
| Flavonoid biosynthesis | 11 | ko00941 |
| MAPK signaling pathway - plant | 10 | ko04016 |
| Carbon fixation in photosynthetic organisms | 9 | ko00710 |
| Glycine, serine and threonine metabolism | 8 | ko00260 |
| Cell cycle | 8 | ko04110 |
| Fluid shear stress and atherosclerosis | 7 | ko05418 |
| Meiosis - yeast | 7 | ko04113 |
| Estrogen signaling pathway | 7 | ko04915 |
| Glyoxylate and dicarboxylate metabolism | 7 | ko00630 |
| Starch and sucrose metabolism | 6 | ko00500 |
| Phenylalanine metabolism | 6 | ko00360 |
| Cell cycle - yeast | 6 | ko04111 |
| Porphyrin and chlorophyll metabolism | 6 | ko00860 |
| Alcoholism | 6 | ko05034 |
| Carotenoid biosynthesis | 6 | ko00906 |
| Pathways in cancer | 6 | ko05200 |
| alpha-Linolenic acid metabolism | 6 | ko00592 |
| Glycolysis / Gluconeogenesis | 6 | ko00010 |
| Fatty acid metabolism | 5 | ko01212 |
| Apoptosis | 5 | ko04210 |
| Epstein-Barr virus infection | 5 | ko05169 |
| Pyrimidine metabolism | 5 | ko00240 |
| Methane metabolism | 5 | ko00680 |
| NOD-like receptor signaling pathway | 5 | ko04621 |
| Viral carcinogenesis | 5 | ko05203 |
| Autophagy - animal | 5 | ko04140 |
| Necroptosis | 5 | ko04217 |
| Insulin signaling pathway | 5 | ko04910 |
| Antigen processing and presentation | 5 | ko04612 |
| cAMP signaling pathway | 5 | ko04024 |
| Amino sugar and nucleotide sugar metabolism | 5 | ko00520 |
| RNA degradation | 5 | ko03018 |
| Oocyte meiosis | 4 | ko04114 |
| GnRH signaling pathway | 4 | ko04912 |
| Pentose and glucuronate interconversions | 4 | ko00040 |
| Thyroid hormone signaling pathway | 4 | ko04919 |
| Ubiquitin mediated proteolysis | 4 | ko04120 |
| Fructose and mannose metabolism | 4 | ko00051 |
| 2-Oxocarboxylic acid metabolism | 4 | ko01210 |
| Ras signaling pathway | 4 | ko04014 |
| Phagosome | 4 | ko04145 |
| Ribosome | 4 | ko03010 |
| Neurotrophin signaling pathway | 4 | ko04722 |
| Proteoglycans in cancer | 4 | ko05205 |
| Biosynthesis of unsaturated fatty acids | 4 | ko01040 |
| Systemic lupus erythematosus | 4 | ko05322 |
| Influenza A | 4 | ko05164 |
| Stilbenoid, diarylheptanoid and gingerol biosynthesis | 4 | ko00945 |
| Oxytocin signaling pathway | 4 | ko04921 |
| Ribosome biogenesis in eukaryotes | 4 | ko03008 |
| GABAergic synapse | 4 | ko04727 |
| Glycerophospholipid metabolism | 4 | ko00564 |
| Ubiquinone and other terpenoid-quinone biosynthesis | 4 | ko00130 |
| DNA replication | 4 | ko03030 |
| Lysosome | 4 | ko04142 |
| Pentose phosphate pathway | 4 | ko00030 |
| Progesterone-mediated oocyte maturation | 4 | ko04914 |
| Cysteine and methionine metabolism | 4 | ko00270 |
| MAPK signaling pathway | 4 | ko04010 |
| Tuberculosis | 4 | ko05152 |
| Glutamatergic synapse | 4 | ko04724 |
| Purine metabolism | 4 | ko00230 |
| Circadian entrainment | 3 | ko04713 |
| Renin secretion | 3 | ko04924 |
| Vascular smooth muscle contraction | 3 | ko04270 |
| Prion diseases | 3 | ko05020 |
| Choline metabolism in cancer | 3 | ko05231 |
| Cellular senescence | 3 | ko04218 |
| Chagas disease | 3 | ko05142 |
| Thyroid hormone synthesis | 3 | ko04918 |
| Melanogenesis | 3 | ko04916 |
| Tryptophan metabolism | 3 | ko00380 |
| Rap1 signaling pathway | 3 | ko04015 |
| Tyrosine metabolism | 3 | ko00350 |
| Pyruvate metabolism | 3 | ko00620 |
| Glutathione metabolism | 3 | ko00480 |
| Phenylalanine, tyrosine and tryptophan biosynthesis | 3 | ko00400 |
| Hepatocellular carcinoma | 3 | ko05225 |
| Prostate cancer | 3 | ko05215 |
| Circadian rhythm - plant | 3 | ko04712 |
| Fatty acid elongation | 3 | ko00062 |
| Isoquinoline alkaloid biosynthesis | 3 | ko00950 |
| Glucagon signaling pathway | 3 | ko04922 |
| AMPK signaling pathway | 3 | ko04152 |
| Long-term potentiation | 3 | ko04720 |
| Toxoplasmosis | 3 | ko05145 |
| Retrograde endocannabinoid signaling | 3 | ko04723 |
| Linoleic acid metabolism | 3 | ko00591 |
| PI3K-Akt signaling pathway | 3 | ko04151 |
| Longevity regulating pathway - multiple species | 3 | ko04213 |
| HTLV-I infection | 3 | ko05166 |
| Adrenergic signaling in cardiomyocytes | 3 | ko04261 |
| Legionellosis | 3 | ko05134 |
| Cyanoamino acid metabolism | 3 | ko00460 |
| Galactose metabolism | 3 | ko00052 |
| Platelet activation | 3 | ko04611 |
| Oxidative phosphorylation | 3 | ko00190 |
| Pertussis | 3 | ko05133 |
| Terpenoid backbone biosynthesis | 3 | ko00900 |
| Human papillomavirus infection | 3 | ko05165 |
| IL-17 signaling pathway | 3 | ko04657 |
| Valine, leucine and isoleucine degradation | 3 | ko00280 |
| Platinum drug resistance | 3 | ko01524 |
| Base excision repair | 3 | ko03410 |
| Peroxisome | 3 | ko04146 |
| Apelin signaling pathway | 3 | ko04371 |
| p53 signaling pathway | 3 | ko04115 |
| Glioma | 2 | ko05214 |
| Spliceosome | 2 | ko03040 |
| Sphingolipid signaling pathway | 2 | ko04071 |
| Antifolate resistance | 2 | ko01523 |
| Adherens junction | 2 | ko04520 |
| Amphetamine addiction | 2 | ko05031 |
| Endocrine resistance | 2 | ko01522 |
| Arginine biosynthesis | 2 | ko00220 |
| Folate biosynthesis | 2 | ko00790 |
| Diterpenoid biosynthesis | 2 | ko00904 |
| Aminoacyl-tRNA biosynthesis | 2 | ko00970 |
| SNARE interactions in vesicular transport | 2 | ko04130 |
| Tropane, piperidine and pyridine alkaloid biosynthesis | 2 | ko00960 |
| Type I diabetes mellitus | 2 | ko04940 |
| Citrate cycle | 2 | ko00020 |
| Gap junction | 2 | ko04540 |
| Longevity regulating pathway - worm | 2 | ko04212 |
| Transcriptional misregulation in cancer | 2 | ko05202 |
| Measles | 2 | ko05162 |
| Monobactam biosynthesis | 2 | ko00261 |
| Phototransduction - fly | 2 | ko04745 |
| Toll-like receptor signaling pathway | 2 | ko04620 |
| Dopaminergic synapse | 2 | ko04728 |
| Chemokine signaling pathway | 2 | ko04062 |
| Central carbon metabolism in cancer | 2 | ko05230 |
| Cytosolic DNA-sensing pathway | 2 | ko04623 |
| Arginine and proline metabolism | 2 | ko00330 |
| ABC transporters | 2 | ko02010 |
| Anthocyanin biosynthesis | 2 | ko00942 |
| MAPK signaling pathway - fly | 2 | ko04013 |
| Fc gamma R-mediated phagocytosis | 2 | ko04666 |
| Phospholipase D signaling pathway | 2 | ko04072 |
| Regulation of actin cytoskeleton | 2 | ko04810 |
| Relaxin signaling pathway | 2 | ko04926 |
| Ascorbate and aldarate metabolism | 2 | ko00053 |
| Gastric acid secretion | 2 | ko04971 |
| Nitrogen metabolism | 2 | ko00910 |
| Endocytosis | 2 | ko04144 |
| Flavone and flavonol biosynthesis | 2 | ko00944 |
| Calcium signaling pathway | 2 | ko04020 |
| NF-kappa B signaling pathway | 2 | ko04064 |
| Vasopressin-regulated water reabsorption | 2 | ko04962 |
| Alzheimer's disease | 2 | ko05010 |
| Wnt signaling pathway | 2 | ko04310 |
| FoxO signaling pathway | 2 | ko04068 |
| Vibrio cholerae infection | 2 | ko05110 |
| Dilated cardiomyopathy | 2 | ko05414 |
| cGMP-PKG signaling pathway | 2 | ko04022 |
| Alanine, aspartate and glutamate metabolism | 2 | ko00250 |
| Bile secretion | 2 | ko04976 |
| Aldosterone synthesis and secretion | 2 | ko04925 |
| Sphingolipid metabolism | 2 | ko00600 |
| Synaptic vesicle cycle | 2 | ko04721 |
| Glycerolipid metabolism | 2 | ko00561 |
| Morphine addiction | 2 | ko05032 |
| Th17 cell differentiation | 2 | ko04659 |
| Serotonergic synapse | 2 | ko04726 |
| beta-Alanine metabolism | 2 | ko00410 |
| Brassinosteroid biosynthesis | 2 | ko00905 |
| Pathogenic Escherichia coli infection | 2 | ko05130 |
| Focal adhesion | 2 | ko04510 |
| Cholinergic synapse | 2 | ko04725 |
| Kaposi's sarcoma-associated herpesvirus infection | 2 | ko05167 |
| Homologous recombination | 2 | ko03440 |
| Inflammatory mediator regulation of TRP channels | 2 | ko04750 |
| Shigellosis | 2 | ko05131 |
| Glucosinolate biosynthesis | 2 | ko00966 |
| Parkinson's disease | 2 | ko05012 |
| Butanoate metabolism | 2 | ko00650 |
| Toll and Imd signaling pathway | 2 | ko04624 |
| PPAR signaling pathway | 2 | ko03320 |
| Salmonella infection | 2 | ko05132 |
| Salivary secretion | 2 | ko04970 |
| Quorum sensing | 2 | ko02024 |
| Ether lipid metabolism | 2 | ko00565 |
| RNA polymerase | 2 | ko03020 |
| Apoptosis - fly | 2 | ko04214 |
| Leishmaniasis | 2 | ko05140 |
| Tight junction | 2 | ko04530 |
| Olfactory transduction | 2 | ko04740 |
| Lysine biosynthesis | 2 | ko00300 |
| TNF signaling pathway | 1 | ko04668 |
| Breast cancer | 1 | ko05224 |
| Bacterial invasion of epithelial cells | 1 | ko05100 |
| Bladder cancer | 1 | ko05219 |
| Other glycan degradation | 1 | ko00511 |
| Zeatin biosynthesis | 1 | ko00908 |
| Biotin metabolism | 1 | ko00780 |
| Viral myocarditis | 1 | ko05416 |
| Protein export | 1 | ko03060 |
| Chemical carcinogenesis | 1 | ko05204 |
| Fatty acid biosynthesis | 1 | ko00061 |
| AGE-RAGE signaling pathway in diabetic complications | 1 | ko04933 |
| Vitamin B6 metabolism | 1 | ko00750 |
| Renin-angiotensin system | 1 | ko04614 |
| B cell receptor signaling pathway | 1 | ko04662 |
| Hypertrophic cardiomyopathy | 1 | ko05410 |
| Gastric cancer | 1 | ko05226 |
| Chlorocyclohexane and chlorobenzene degradation | 1 | ko00361 |
| Leukocyte transendothelial migration | 1 | ko04670 |
| One carbon pool by folate | 1 | ko00670 |
| Prolactin signaling pathway | 1 | ko04917 |
| Endocrine and other factor-regulated calcium reabsorption | 1 | ko04961 |
| Riboflavin metabolism | 1 | ko00740 |
| Ovarian steroidogenesis | 1 | ko04913 |
| Aldosterone-regulated sodium reabsorption | 1 | ko04960 |
| Signaling pathways regulating pluripotency of stem cells | 1 | ko04550 |
| Thiamine metabolism | 1 | ko00730 |
| Rheumatoid arthritis | 1 | ko05323 |
| Circadian rhythm | 1 | ko04710 |
| Hepatitis B | 1 | ko05161 |
| Th1 and Th2 cell differentiation | 1 | ko04658 |
| Long-term depression | 1 | ko04730 |
| T cell receptor signaling pathway | 1 | ko04660 |
| Toluene degradation | 1 | ko00623 |
| Type II diabetes mellitus | 1 | ko04930 |
| Longevity regulating pathway | 1 | ko04211 |
| Monoterpenoid biosynthesis | 1 | ko00902 |
| Pantothenate and CoA biosynthesis | 1 | ko00770 |
| Cocaine addiction | 1 | ko05030 |
| Isoflavonoid biosynthesis | 1 | ko00943 |
| Chronic myeloid leukemia | 1 | ko05220 |
| Glycosphingolipid biosynthesis - globo and isoglobo series | 1 | ko00603 |
| MicroRNAs in cancer | 1 | ko05206 |
| Fluorobenzoate degradation | 1 | ko00364 |
| Thyroid cancer | 1 | ko05216 |
| Caffeine metabolism | 1 | ko00232 |
| Insulin secretion | 1 | ko04911 |
| Biofilm formation - Escherichia coli | 1 | ko02026 |
| Osteoclast differentiation | 1 | ko04380 |
| Novobiocin biosynthesis | 1 | ko00401 |
| Hippo signaling pathway | 1 | ko04390 |
| mTOR signaling pathway | 1 | ko04150 |
| Cutin, suberine and wax biosynthesis | 1 | ko00073 |
| Metabolism of xenobiotics by cytochrome P450 | 1 | ko00980 |
| Phosphatidylinositol signaling system | 1 | ko04070 |
| Pancreatic cancer | 1 | ko05212 |
| Colorectal cancer | 1 | ko05210 |
| Acute myeloid leukemia | 1 | ko05221 |
| Hedgehog signaling pathway | 1 | ko04340 |
| Non-small cell lung cancer | 1 | ko05223 |
| Benzoxazinoid biosynthesis | 1 | ko00402 |
| Glycosylphosphatidylinositol | 1 | ko00563 |
| Endometrial cancer | 1 | ko05213 |
| Drug metabolism - other enzymes | 1 | ko00983 |
| Hepatitis C | 1 | ko05160 |
| Regulation of lipolysis in adipocytes | 1 | ko04923 |
| Axon guidance | 1 | ko04360 |
| ErbB signaling pathway | 1 | ko04012 |
| Melanoma | 1 | ko05218 |
| HIF-1 signaling pathway | 1 | ko04066 |
| Taurine and hypotaurine metabolism | 1 | ko00430 |
| Hedgehog signaling pathway - fly | 1 | ko04341 |
| Dorso-ventral axis formation | 1 | ko04320 |
| Valine, leucine and isoleucine biosynthesis | 1 | ko00290 |
| Biosynthesis of ansamycins | 1 | ko01051 |
| Two-component system | 1 | ko02020 |
| VEGF signaling pathway | 1 | ko04370 |
| Proteasome | 1 | ko03050 |
| Drug metabolism - cytochrome P450 | 1 | ko00982 |
| TGF-beta signaling pathway | 1 | ko04350 |
| Arrhythmogenic right ventricular cardiomyopathy | 1 | ko05412 |
| MAPK signaling pathway - yeast | 1 | ko04011 |
| Nicotine addiction | 1 | ko05033 |
| Mineral absorption | 1 | ko04978 |
| Phototransduction | 1 | ko04744 |
| Autophagy - yeast | 1 | ko04138 |
| Hippo signaling pathway - fly | 1 | ko04391 |
| Arachidonic acid metabolism | 1 | ko00590 |
| Natural killer cell mediated cytotoxicity | 1 | ko04650 |
| Insulin resistance | 1 | ko04931 |
| Prodigiosin biosynthesis | 1 | ko00333 |
| Propanoate metabolism | 1 | ko00640 |
| Renal cell carcinoma | 1 | ko05211 |
| Fc epsilon RI signaling pathway | 1 | ko04664 |
| EGFR tyrosine kinase inhibitor resistance | 1 | ko01521 |
| Degradation of aromatic compounds | 1 | ko01220 |
| Taste transduction | 1 | ko04742 |
| Amoebiasis | 1 | ko05146 |
| Fatty acid degradation | 1 | ko00071 |
